# Regression of prostate tumors after intravenous administration of lactoferrin-bearing polypropylenimine dendriplexes encoding TNF-α, TRAIL, and interleukin-12

**DOI:** 10.1080/10717544.2018.1440666

**Published:** 2018-03-01

**Authors:** Najla Altwaijry, Sukrut Somani, John A. Parkinson, Rothwelle J. Tate, Patricia Keating, Monika Warzecha, Graeme R. Mackenzie, Hing Y. Leung, Christine Dufès

**Affiliations:** a Strathclyde Institute of Pharmacy and Biomedical Sciences, University of Strathclyde, Glasgow, UK;; b Department of Pure and Applied Chemistry, University of Strathclyde, Glasgow, UK;; c Cancer Research UK Beatson Institute, Glasgow, UK

**Keywords:** Prostate cancer, gene therapy, dendrimer, lactoferrin, nanoparticles

## Abstract

The possibility of using gene therapy for the treatment of prostate cancer is limited by the lack of intravenously administered delivery systems able to safely and selectively deliver therapeutic genes to tumors. Given that lactoferrin (Lf) receptors are overexpressed on prostate cancer cells, we hypothesized that the conjugation of Lf to generation 3-diaminobutyric polypropylenimine dendrimer would improve its transfection and therapeutic efficacy in prostate cancer cells. In this study, we demonstrated that the intravenous administration of Lf-bearing DAB dendriplexes encoding TNFα resulted in the complete suppression of 70% of PC-3 and 50% of DU145 tumors over one month. Treatment with DAB-Lf dendriplex encoding TRAIL led to tumor suppression of 40% of PC-3 tumors and 20% of DU145 tumors. The treatment was well tolerated by the animals. Lf-bearing generation 3-polypropylenimine dendrimer is therefore a highly promising delivery system for non-viral gene therapy of prostate cancer.

## Introduction

Prostate cancer is the fourth most widespread cancer in the world, the second most common cancer in men, and the first in Europe and North America (Ferlay et al., 2015). It is responsible of the death of 300 000 patients per year worldwide and its incidence kept on increasing during the last two decades (Saman et al., 2014). Although cryoablation, chemotherapy, radiotherapy, and radical prostatectomy can be efficacious against localized tumors, there is still no effective treatment for patients with recurrent or metastatic disease (Lu, 2009). New therapeutic approaches are therefore urgently needed for these patients.

Among novel experimental strategies, gene therapy would be highly promising for the treatment of prostate cancer, but its use is currently limited by the lack of delivery systems able to selectively deliver the therapeutic genes to the tumors following intravenous administration, without creating secondary effects to healthy tissues.

In order to remediate this issue, we propose to conjugate the iron-carrier lactoferrin (Lf), whose receptors are overexpressed on prostate cancer cell lines (Barresi & Tuccari, 1984; Tuccari & Barresi, 2011), to the surface of generation 3-diaminobutyric polypropylenimine (DAB) dendrimer carrying plasmids encoding for TNFα, TRAIL, or IL-12, and evaluate if these Lf-bearing dendriplexes could lead to an enhanced therapeutic efficacy on prostate cancer following intravenous administration.

Lactoferrin (Lf) is an iron-binding member of the transferrin family, which has been shown to have intrinsic anti-tumoral activity, making it particularly attractive as part of a gene medicine. Lf binds to specific receptors (Lf R1, Lf R2) or to transferrin receptors overexpressed on most cancers lines (Barresi & Tuccari, 1984; Tuccari & Barresi, 2011). It has been shown to exert its anticancer effect through modulation of the mitogen-activated protein kinase signaling pathway and induction of cell cycle arrest, and can also induce apoptosis of cancer cells by activating the Fas signaling pathway in cancerous cells (Zhou et al., 2008; Gibbons et al., 2011).

TNFα, an inflammatory cytokine, is well known for its ability to induce apoptosis in a wide variety of cancer cells, including prostate cancer cells. Fas-sensitive PC-3 prostate cancer cells were reported to be more sensitive to TNFα-mediated apoptosis and growth inhibition than Fas-resistant DU145 and LNCaP cells (Rokhlin et al., 1997). In addition, TNF-α is able to act on the tumor-associated vasculature by inducing hyperpermeability and destruction of the vascular lining.

TRAIL, a member of the TNF family of cytokines, presents the particularly promising advantage to induce apoptosis preferentially in cancer cells whilst sparing normal cells (van Ophoven et al., 1999). Its binding to death receptors DR4 and DR5 resulted in the recruitment of Fas-associated death domain, and cancer cell death via caspase-dependent apoptotic pathways (van Ophoven et al., 1999).

Another cytokine, IL-12, exerts its anti-cancer effect using various mechanisms such as activation of potent antitumor immunity, induction of interferon-gamma, interferon-inducible protein-10 (IP-10), and regulation of Th1 cell differentiation (Gately et al., 1998; Tannenbaum et al., 1998). In animal melanoma models, the administration of IL-12 plasmid resulted in inhibition of tumor growth and regression of established tumors (Lucas et al., 2002), but is also limited by its potential side effects (Leonard et al., 1997), thus highlighting the need of a tumor-targeted delivery system for delivering this plasmid to its site of action.

The objectives of this study were therefore (1) to synthesize and characterize Lf-bearing DAB dendrimer, (2) to assess the transfection and anti-proliferative efficacy of the DAB-Lf dendriplexes encoding TNFα, TRAIL, or IL-12, on prostate cancer cells *in vitro*, and (3) to evaluate their therapeutic efficacy *in vivo*, following intravenous administration to mice bearing prostate tumors.

## Methods

### Cell lines and reagents

Generation 3-diaminobutyric polypropylenimine dendrimer (DAB), Lf, and the other chemicals were purchased from Sigma Aldrich (Poole, UK). The cross-linker N-γ-maleimidobutyryl-oxysuccinimide ester (GMBS) was from PolyPeptide (Strasbourg, France). The expression plasmids encoding β-galactosidase (pCMVsport β-galactosidase) and TNFα (pORF9-mTNFα) were obtained respectively from Invitrogen (Paisley, UK) and InvivoGen (San Diego, CA), the expression plasmids encoding TRAIL (pORF9-mTRAIL) and IL-12 (pORF-mIL12) came from Source BioScience (Nottingham, UK). All these plasmids were purified using an Endotoxin-free Giga Plasmid Kit (Qiagen, Hilden, Germany). Quant-iT™ PicoGreen^®^ dsDNA reagent, AlexaFluor^®^ 647 phalloidin probes and tissue culture media were purchased from Life Technologies (Paisley, UK). Vectashield^®^ mounting medium containing 4′,6-diamino-2-phenylindole (DAPI) was obtained from Vector Laboratories (Peterborough, UK). Label IT^®^ Fluorescein Nucleic Acid Labeling Kit was from Cambridge Biosciences (Cambridge, UK). Passive lysis buffer was obtained from Promega (Southampton, UK). AgarGel H/M agarose was from Continental Laboratory Products (Northampton, UK). HyperLadder I was from Bioline (London, UK). Firefly D-luciferin and Bioware^®^ androgen-irresponsive PC-3 M-luc-C6 human prostate adenocarcinoma that expresses the firefly luciferase were from Caliper Life Sciences (Hopkinton, MA), while androgen-irresponsive DU145 human prostate carcinoma was purchased from the European Collection of Cell Cultures (Salisbury, UK).

### Synthesis and characterization of Lf-bearing DAB (in supplementary material)

Generation 3-diaminobutyric polypropylenimine dendrimer (DAB) was conjugated to Lf with GMBS as a cross-linking agent, using a method adapted from Hermanson (2013) (Supplementary Figure 1). The grafting of Lf to DAB was assessed by ^1 ^H NMR spectroscopy (Jeol Oxford NMR AS 600 spectrometer, Peobody, MA) and MALDI-TOF mass spectroscopy (Axima CFR, Kratos, Shimadzu, Kyoto, Japan).

### *In vitro* biological characterization

#### Cell culture

PC-3 and DU145 cells were grown as monolayers in Minimum Essential Medium (MEM) supplemented with 10% (v/v) fetal bovine serum, 1% (v/v) L-glutamine, and 0.5% (v/v) penicillin–streptomycin. The cell culture flasks were kept at 37 °C in a 5% carbon dioxide, humid atmosphere.

#### *In vitro* transfection

The transfection efficacy of the DNA complexed by DAB-Lf was assessed using a plasmid DNA encoding β-galactosidase. PC-3 and DU145 cells were seeded at a concentration of 2000 cells/well in 96-well plates and incubated for 72 h at 37 °C in a 5% CO_2_ atmosphere. They were then treated with DAB-Lf dendriplex in quintuplicate at various dendrimer: DNA weight ratios (20:1, 10:1, 5:1, 2:1, 1:1, and 0.5:1). Naked DNA was used as a negative control and DAB-DNA (dendrimer: DNA weight ratio 5:1) served as a positive control. DNA concentration (10 µg/mL) was kept constant throughout the experiment. After treatment, the cells were incubated for 72 h before analysis. They were then lysed with 1× passive lysis buffer (PLB) (50 µL/well) for 20 min and then tested for β-galactosidase expression (Zinselmeyer et al., 2002). Briefly, 50 µL of the assay buffer (2 mM magnesium chloride, 100 mM mercaptoethanol, 1.33 mg/mL O-nitrophenyl- β-D-galactosidase, 200 mM sodium phosphate buffer, pH 7.3) was added in each well containing the lysates, before being incubated for 2 h at 37 °C. The absorbance of the samples was subsequently read at 405 nm using a Multiskan Ascent^®^ plate reader (MTX Lab Systems, Brandenton, FL).

#### Cellular uptake

##### Qualitative analysis

The cellular uptake of DAB-Lf dendriplex was qualitatively assessed using confocal microscopy. Labeling of plasmid DNA with the fluorescent probe fluorescein was performed using a Label IT^®^ Fluorescein Nucleic Acid Labeling kit, as described by the manufacturer. PC-3 and DU145 cells were seeded on coverslips in 6-well plates at a concentration of 10^5^ cells per well and grown for 24 h at 37 °C. The cells were then treated with fluorescein-labeled DNA (2.5 µg/well) complexed to DAB-Lf and DAB at a dendrimer: DNA weight ratio of 5:1 for 24 h at 37 °C. Control wells were also prepared with naked DNA or remained untreated.

The cells were then washed three times with 3 mL phosphate-buffered saline (PBS) before being fixed with 3 mL formaldehyde solution (3.7%) for 10 min at 20 °C. They were then washed again with 3 mL PBS, incubated at 20 °C with 3 mL Triton-X100 solution (0.1%) for 5 min, before a further incubation with 3 mL of bovine serum albumin solution (1% w/v in PBS) for 30 min at 37 °C. One unit of Alexa Fluor^®^ 647 (in 200 µL PBS) was then added to the wells, incubated for 20 min at 20 °C, before a final wash with 3 mL PBS. Upon staining of the nuclei with Vectashield^®^ mounting medium containing DAPI, the cells were examined using a Leica TCS SP5 confocal microscope (Wetzlar, Germany). DAPI (which stained the cell nuclei) was excited with the 405 nm laser line (emission bandwidth: 415–491 nm), fluorescein (which labeled the DNA) was excited with the 514 nm laser line (emission bandwidth: 550–620 nm), whereas Alexa Fluor^®^ 647 (which stained the cell wall) was excited with the 633 nm laser line (emission bandwidth: 650–690 nm).

##### Quantitative analysis

Quantification of cellular uptake of DAB-Lf dendriplex was carried out by flow cytometry. PC-3 and DU145 cells were seeded at a density of 1.6 × 10^5^ cells per well in six-well plates and grown at 37 °C for 72 h. The cells were then treated with fluorescein-labeled DNA (5 µg DNA/well) complexed with DAB-Lf at a dendrimer: DNA weight ratio of 5:1. Other wells were treated with DAB dendriplex and DNA solution as positive and negative controls, respectively.

After 24 h incubation with the treatments, single cell suspensions were prepared (using 250 µL trypsin per well, followed by 500 µL medium per well once the cells have detached), before being analyzed using a FACSCanto^®^ flow cytometer (BD, Franklin Lakes, NJ). Ten thousand cells (gated events) were counted for each sample. Their mean fluorescence intensity was analyzed with FACSDiva^®^ software (BD, Franklin Lakes, NJ).

##### Mechanisms of cellular uptake of DNA complexed to DAB-Lf

The mechanisms involved in the cellular uptake of DNA complexed to DAB-Lf were investigated using various uptake inhibitors. PC-3 and DU145 cells were seeded in six-well plates (1 × 10^5^ cells per well) and incubated at 37 °C for 24 h for qualitative analysis or 72 h for quantitative analysis.

After removal of the medium, they were pretreated with free Lf (80 μM), phenylarsine oxide (10 µmol/L), filipin (5 µg/mL), colchicine (10 µmol/L), and poly-L-lysine (40 µg/mL) for 15 min at 37 °C. The cells were then treated with fluorescein-labeled DNA (respectively, 2.5 and 5 μg/well for qualitative and quantitative analysis) complexed to DAB-Lf (dendrimer: DNA weight ratio of 5:1) for 1 h at 37 °C, before being washed and processed for confocal microscopy and flow cytometry analysis as described above.

### *In vitro* antiproliferative activity

The antiproliferative activity of Lf-bearing DAB complexed with plasmid DNA encoding TNFα, TRAIL, or IL-12 was assessed using a standard MTT assay. PC-3 and DU145 cells were seeded in quintuplicate at a density of 2000 cells/well in 96-well plates for 72 h before treatment. They were then incubated for 72 h with the DAB-Lf dendriplex formulations at final DNA concentrations ranging from 0.05 to 200 µg/mL, using naked DNA as a negative control, while DAB: DNA 5:1 ratio served as a positive control. Absorbance was measured at 570 nm using a plate reader and the growth inhibitory concentration for 50% of the cells (IC_50_) was measured. Dose-response curves were fitted to percentage absorbance values to obtain IC_50_ values (three independent experiments, each performed in quintuplicate).

### *In vivo* study

The *in vivo* experiments were approved by the local ethics committee and performed in accordance with the UK Home Office regulations.

PC-3 M-luc-C6 and DU145 cancer cells in exponential growth were subcutaneously implanted to both flanks of male immunodeficient BALB/c mice (1 × 10^6^ cells per flank). When tumors became palpable, vascularized, and reached a diameter of 5 mm, the animals were split into groups of five and were treated with DAB-Lf dendriplexes encoding TNFα, TRAIL, or IL-12, the targeted dendrimer and naked DNA (50 µg of DNA), via tail vein injection, once every 2 days for 10 days. The weight of the mice was measured daily as a surrogate marker of the toxicity of the treatments and tumor volume was determined by caliper measurements (volume = *d*^3^ × π/6). Any animal showing a weight loss of 20% or more of its initial body weight (which demonstrates toxicity of the treatment) would have to be humanely killed.

The therapeutic efficacy of these treatments was also assessed by bioluminescence imaging, using an IVIS Spectrum (Caliper Life Sciences, Hopkinton, MA). To this end, mice bearing subcutaneous PC-3 M-luc-C6 tumors were intravenously injected with treatments as described above. Ten minutes before imaging, they were intraperitoneally injected with the luciferase substrate D-luciferin (150 mg/kg body weight), and then anesthetized by isoflurane inhalation on Days 1, 3, 5, 7, 9 of the experiment. The light emitted from the bioluminescent tumors was detected for 2 min using Living Image^®^ software and displayed as a pseudo-color overlay onto a gray scale image of the animal. Identical illumination settings were used for acquiring all images.

### Statistical analysis

Results were expressed as means ± standard error of the mean. Statistical significance was assessed by one-way analysis of variance and Tukey multiple comparison post-test (Minitab^®^ software, State College, PE). Differences were considered statistically significant for *p* values lower than .05.

## Results

### Synthesis and characterization of Lf-bearing DAB (in supplementary material) (supplementary figures 2–4)

#### *In vitro* biological characterization

##### *In vitro* transfection

**Figure 1.**
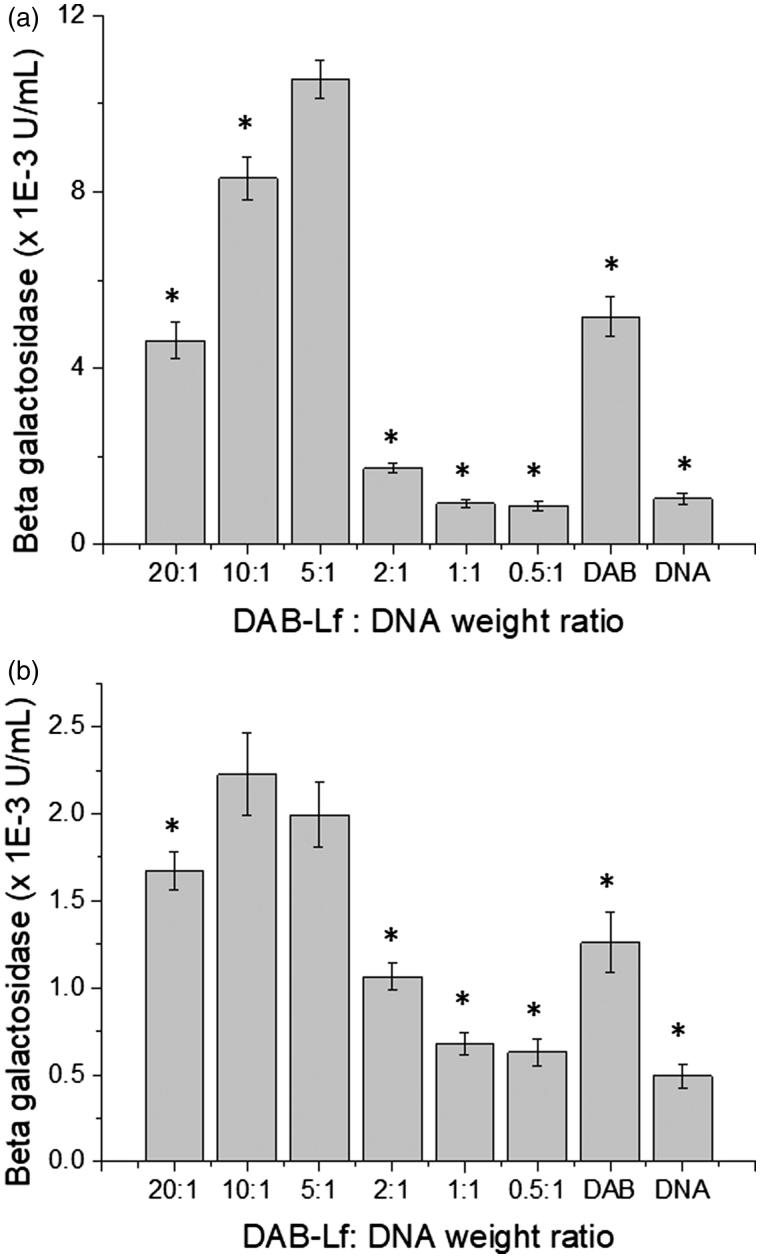
Transfection efficacy of DAB-Lf dendriplex at various dendrimer: DNA weight ratios in PC-3 (a) and DU145 cells (b). Results are expressed as the mean ± SEM of three replicates (*n* = 15). *p < .05 versus the highest transfection ratio.

Lf-bearing DAB dendrimer was found to increase gene transfection at optimal dendrimer: DNA weight ratios compared with DAB on both the tested cell lines (Figure 1). The highest transfection level was obtained at dendrimer: DNA weight ratios of 5:1 on PC-3 (Figure 1(a)), 5:1 and 10:1 on DU145 cells (Figure 1(b)). At these ratios, gene expression following treatment with DAB-Lf dendriplex (10.54 × 10^−3^ ± 0.43 × 10^−3 ^U/mL) was 2-fold higher than following treatment with DAB dendriplex (5.17 × 10^−3^ ± 0.46 × 10^−3 ^U/mL) in PC3 cells. In DU145 cells, transfection efficacy of DAB-Lf dendriplex (respectively, 2.23 × 10^−3^ ± 0.23 × 10^−3 ^U/mL and 1.99 × 10^−3^ ± 0.18 × 10^−3 ^U/mL for dendrimer: DNA weight ratios of 10:1 and 5:1) was lower than that observed in PC3 cells, but was still significantly higher than that observed with DAB dendriplex (1.26 × 10^−3^ ± 0.17 × 10^−3^). Treatment with naked DNA only resulted in weak levels of gene expression on both cell lines, as expected.

##### Cellular uptake

**Figure 2.**
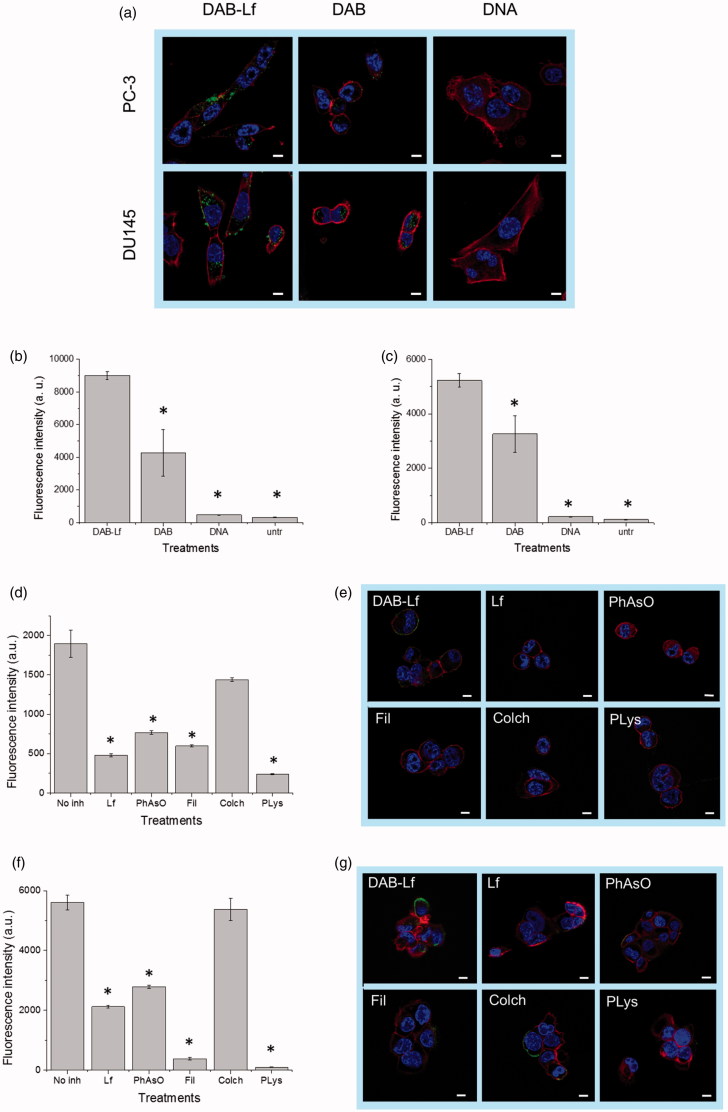
Cellular uptake of fluorescein-labeled DNA complexed with DAB-Lf in PC-3 and DU145 cells: (a) Confocal microscopy imaging of the cellular uptake of fluorescein-labeled DNA (2.5 μg/well) complexed with DAB-Lf, DAB or left uncomplexed, after incubation for 24 h with PC-3 and DU145 cells (Blue: nuclei stained with DAPI (excitation: 405 nm laser line, bandwidth: 415–491 nm), green: fluorescein-labeled DNA (excitation: 543 nm laser line. bandwidth: 550–620 nm), red: Alexa Fluor^®^ 647 probe (excitation: 633 nm laser line, bandwidth: 650–690 nm) (Bar: 10 µm). (b) Flow cytometry quantification of the cellular uptake of fluorescein-labeled DNA (5 µg/well) either complexed with DAB-Lf, DAB or left uncomplexed, after incubation for 24 h with PC-3 cells (*n* = 5). *p < .05 compared with DAB-Lf-DNA. (c) As in B, but with DU145 cells. (d) Flow cytometry quantification of the PC-3 cellular uptake of fluorescein- labeled DNA (5 µg/well) complexed with DAB-Lf, following pretreatment with free Lf (80 µM) and various cellular uptake inhibitors: phenylarsine oxide (‘PhAsO’), filipin (‘Fil.’), colchicine (‘Colch.’) and poly-L-lysine (‘PLys’) (*n* = 5), *p < .05 compared with DAB-Lf-DNA. (e) Confocal microscopy imaging of the PC-3 cellular uptake of fluorescein-labeled DNA (2.5 μg/well) complexed with DAB-Lf, following pretreatment with free Lf (80 µM) and various cellular uptake inhibitors: phenylarsine oxide (‘PhAsO’), filipin (‘Fil.’), colchicine (‘Colch.’) and poly-L-lysine (‘PLys’) (Blue: nuclei stained with DAPI (excitation: 405 nm laser line, bandwidth: 415–491 nm), green: fluorescein-labeled DNA (excitation: 543 nm laser line. bandwidth: 550–620 nm), red: Alexa Fluor^®^ 647 probe (excitation: 633 nm laser line, bandwidth: 650–690 nm) (Bar: 10 µm). (f) As in d, but with DU145 cells (g) As in e, but with DU145 cells.

*Qualitative and quantitative analysis.* The cellular uptake of fluorescein-labeled DNA carried by DAB-Lf was qualitatively and quantitatively confirmed in the two prostate cancer cell lines by confocal microscopy and flow cytometry (Figure 2). Fluorescein-labeled DNA was disseminated in the cytoplasm after treatment with both DAB-Lf and DAB dendriplexes in PC-3 and DU145 cells. However, the DNA uptake appeared to be more pronounced in the cells treated with DAB-Lf dendriplex than with DAB dendriplex. No co-localization of DNA in the nuclei was visible in any cell lines after 24 h incubation (Figure 2(a)).

These results were quantitatively confirmed by flow cytometry. Treatment of PC3 cells with DAB-Lf dendriplex resulted in the highest cellular fluorescence (9004 ± 241 arbitrary units (a.u.)), which was, respectively, 2.1-fold and 19.2-fold higher than that observed after treatment with DAB dendriplex (4 266 ± 1 420 a.u.) and DNA solution (469 ± 22 a.u.) (Figure 2(b)). Similarly, the highest cellular fluorescence in DU145 cells was also observed following treatment with DAB-Lf dendriplex (5 227 ± 251 a.u.). It was, respectively, 1.6-fold and 23.8-fold higher than that observed following treatment with DAB dendriplex (3 261 ± 677 a.u.) and DNA solution (219 ± 12 a.u.) (Figure 2(c)). Furthermore, treatment of the cells with naked DNA resulted in a weak uptake for both cell lines, thus demonstrating the failure of DNA to be taken up by prostate cancer cells without the assistance of a carrier.

*Mechanisms of cellular uptake of DNA complexed to DAB-Lf.* Pre-treatment of PC-3 cells with 80 µM of free Lf significantly decreased the cellular uptake of fluorescein-labeled DNA complexed to DAB-Lf, which was 3.9-fold lower than that observed with DAB-Lf dendriplex without pre-Lf treatment (respectively, 482 ± 20 a. u. and 1 897 ± 173 a. u.) (Figures 2(d,e)). The cellular uptake of fluorescein-labeled DNA complexed to DAB-Lf was also partially inhibited by phenylarsine oxide, filipin, colchicine, and poly-L-lysine in PC-3 cells (Figures 2(d,e)). Poly-L-lysine caused the most significant inhibition, with a cellular uptake decreased by 7.9-fold compared to that observed with DAB-Lf dendriplex without inhibitory treatment (239 ± 8 a.u. following pretreatment with poly-L-lysine). Phenylarsine oxide and filipin appear to be partial inhibitors, leading to a cellular uptake decrease by, respectively, 2.4-fold and 3.1-fold compared to DAB-Lf dendriplex without pretreatment (respectively, 766 ± 22 a. u. and 597 ± 13 a.u. following pretreatment with phenylarsine oxide and filipin). Colchicine caused the least inhibition with 1.3-fold decrease in the cellular uptake (1437 ± 22 a.u following pretreatment with colchicine).

The uptake of fluorescein-labeled DNA complexed to DAB-Lf by DU145 cells was also partially inhibited by free Lf, phenylarsine oxide, filipin, colchicine, and poly-L-lysine, following the same inhibition pattern than that observed in PC-3 cells (Figures 2(f,g)). Poly-L-lysine caused the most significant inhibition, with a cellular uptake decreased by 57.8-fold compared to that observed with DAB-Lf dendriplex (respectively, 97 ± 3 a.u. following pretreatment with poly-L-lysine and 5 607 ± 248 a.u. following treatment with DAB-Lf dendriplex). Colchicine also caused the least inhibition with a non-significantly different decrease in the cellular uptake (5374 ± 371 a.u following pretreatment with colchicine). Phenylarsine oxide and filipin were on this cell line as well partial inhibitors (respectively, 2791 ± 53 a. u. and 381 ± 37 a.u. following pretreatment with phenylarsine oxide and filipin, corresponding to a cellular uptake decrease by, respectively, 2-fold and 14.7-fold compared to DAB-Lf dendriplex).

#### *In vitro* anti-proliferative activity

The conjugation of Lf to DAB complexed to TNFα, TRAIL, and IL-12 expression plasmids led to a significant increase of *in vitro* antiproliferative activity in PC-3, respectively by 1.9-fold, 3.5-fold, and 5.8-fold for DAB-Lf dendriplexes encoding TNFα, TRAIL and IL12 when compared to the unconjugated dendriplex (Table 1, Supplementary Figure 5). DAB-Lf dendriplex encoding TNFα was the most efficacious (IC_50_: 9.97 ± 1.38 µg/mL), followed by the dendriplex encoding IL-12 (IC_50_: 10.84 ± 2.71 µg/mL), and then the dendriplex encoding TRAIL (IC_50_: 15.62 ± 3.61 µg/mL). The maximum inhibition of proliferation was reached at a DNA concentration of 80 µg/mL, with 11.9 ± 0.6, 25.3 ± 1.5, and 13.2 ± 0.6% of cells still alive following treatment with DAB-Lf dendriplexes encoding TNFα, TRAIL, and IL12, respectively.

**Table 1.** Antiproliferative efficacy of DAB-Lf dendriplexes encoding TNFα, TRAIL, and IL-12, expressed as IC_50_ values, in PC-3 and DU145 prostate cancer cells (n.d.: not determined) (*n* = 15).

IC_50_ (µg/mL) (mean ± SEM)						
Formulation						
Cell line	DNA	DAB-Lf-DNA	DAB-DNA	DAB-Lf only	DAB only	DNA only
PC-3	TNFα	9.97 ± 1.38	19.50 ± 3.44	n.d.	n.d.	n.d.
	TRAIL	15.62 ± 3.61	55.74 ± 22.36	n.d.	n.d.	n.d.
	IL-12	10.84 ± 2.71	63.33 ± 37.89	n.d.	n.d.	n.d.
DU145	TNFα	4.34 ± 0.68	57.38 ± 95.43	n.d.	n.d.	n.d.
	TRAIL	3.03 ± 0.70	17.14 ± 3.78	n.d.	n.d.	n.d.
	IL-12	3.80 ± 0.97	5.92 ± 0.96	n.d.	n.d.	n.d.

In DU145 cells, the conjugation of Lf to DAB also improved the anti-proliferative activity of the dendriplex, by 13.2-fold, 5.6-fold, and 1.5-fold for DAB-Lf dendriplexes encoding TNFα (IC_50_: 4.34 ± 0.68 µg/mL), TRAIL (IC_50_: 3.03 ± 0.70 µg/mL), and IL-12 (IC_50_: 3.80 ± 0.97 µg/mL) (Table 1, Supplementary Figure 6). The IC_50_ could not be determined following treatment of the cells with uncomplexed DAB-Lf, DAB, and naked DNA. The inhibition of proliferation was almost complete at a DNA concentration of 32 µg/mL, with 5.8 ± 1.3, 4.3 ± 0.6, and 3.9 ± 0.1% of cells still alive following treatment with DAB-Lf dendriplexes encoding TNFα, TRAIL and IL12, respectively.

## *In vivo* study

**Figure 3.**
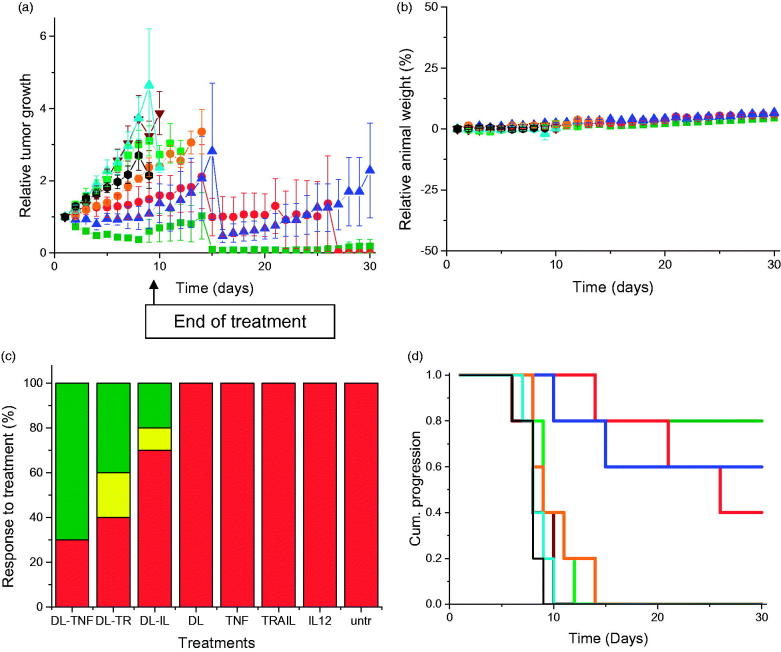
(a) Tumor growth studies in a PC-3 xenograft model after intravenous administration of DAB-Lf dendriplex encoding TNFα (▪, green), TRAIL (•, red), IL-12 (▲, blue) (50 µg DNA/injection), DAB-Lf (▼, brown), naked DNA encoding TNFα (▪, pale green), naked DNA encoding TRAIL (•, orange), naked DNA encoding IL-12 (▲, cyan), untreated tumors (•, back) (*n* = 5). (b) Variations of the animal body weight throughout the treatment (Color coding as in a) (*n* = 5). (c) Overall tumor response to treatments at the end of the study, classified in accordance with the Response Evaluation Criteria in Solid Tumors (RECIST) (Eisenauer et al., 2009) (red: progressive response, orange: stable response, yellow: partial response, green: complete response) (*n* = 5). (d) Time to disease progression. The Y axis gives the proportion of surviving animals over time. Animals were removed from the study once their tumor reached 10 mm diameter (Color coding as in a) (*n* = 5).

**Figure 4.**
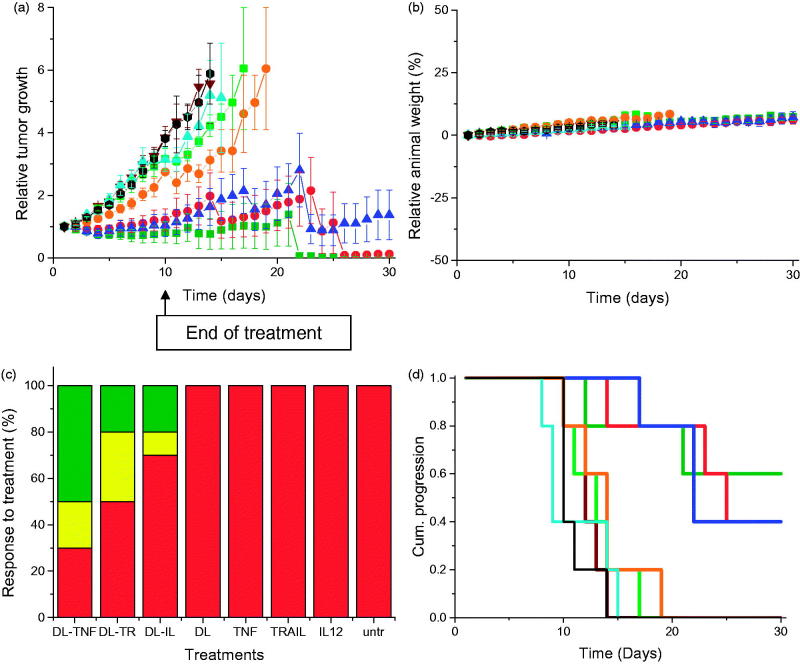
As in Figure 3, but in DU145 cells.

Treatment of PC-3 tumors with the three dendriplex formulations (DAB-Lf dendriplexes encoding TNFα, TRAIL, or IL12) was characterized by a high variability of response to treatment within the same group of mice and an overall reduced tumor growth compared to naked DNA treatment (Figure 3a). For all of these three treatments, some tumors kept regressing while others started growing. At Days 14 and 15, the mice bearing growing tumors had to be euthanized due to their tumors reaching the maximum allowed size. The remaining mice, whose tumors were regressing or had completely disappeared, were kept until the end of the study (Day 30). By contrast, tumors treated with naked DNA grew steadily at a growth rate close to that observed for untreated tumors. Treatment of the DU145 tumors with the three dendriplex formulations led to a similar pattern of overall slowdown of tumor growth (Figure 4(a)).

**Figure 5.**
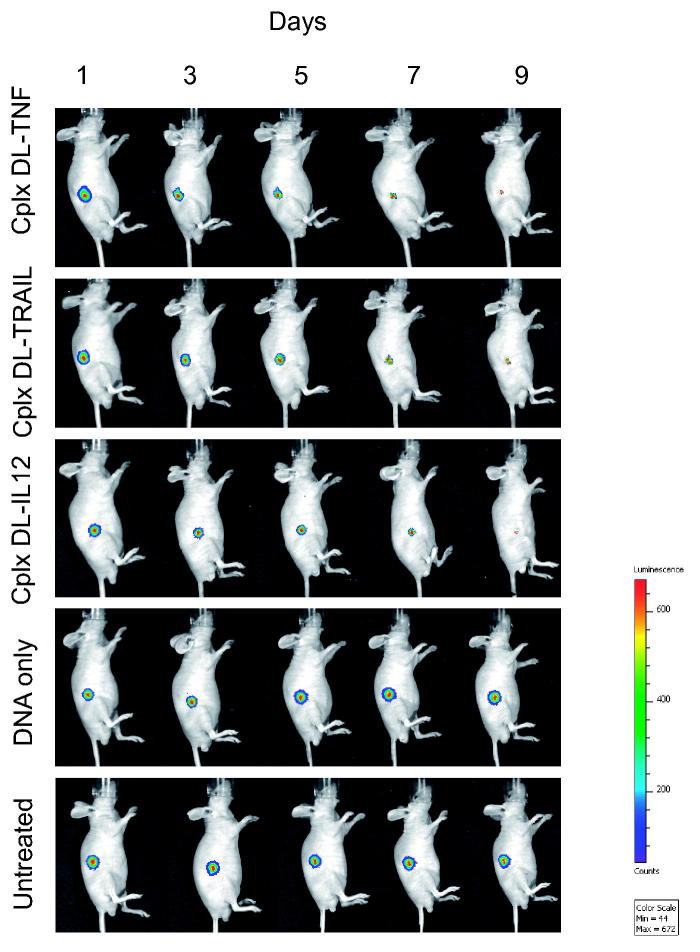
Bioluminescence imaging of the tumoricidal activity of DAB-Lf dendriplex encoding TNFα (‘cplx DT-TNF’), TRAIL (‘cplx DT-TRAIL’), IL-12 (‘cplx DT-IL12’) in a PC-3 M-luc-C6 tumor model. Controls: naked DNA and untreated tumors. The scale indicates surface radiance (photons/s/cm^2^/steradian).

The therapeutic effect resulting from treatment with DAB-Lf dendriplexes was also qualitatively confirmed by bioluminescence imaging on mice bearing subcutaneous PC-3 M-luc-C6 tumors (Figure 5). Luciferase expression in the tumors treated with the three formulations of DAB-Lf dendriplexes gradually decreased from Days 1 to 9, whereas all the other treatments resulted in an increase of luciferase expression in the growing tumors.

No animal weight loss or apparent signs of toxicity (marked dyspnea, vocalization, prostration, paleness, hunched posture, lack of food, or water intake) were observed during the whole study, thus showing the good tolerability of the treatments by the mice (Figures 3(b) and 4(b)).

On the last day of the experiment, 70% of PC-3 and 50% of DU145 tumors treated with DAB-Lf dendriplex encoding TNFα had completely disappeared, while another 10% of DU145 tumors showed a partial response (Figures 3(c) and 4(c)). Following treatment with DAB-Lf dendriplex encoding TRAIL, 40% of PC-3 tumors and 20% of DU145 tumors showed a complete disappearance, 10% of PC-3 and 30% of DU145 tumors were regressing. Finally, treatment with DAB-Lf dendriplex encoding IL-12 resulted in tumor disappearance of 20% of both PC-3 and DU145 tumors and tumor regression of 10% on both tumor types. All the tumors treated with naked DNA or left untreated were progressive for both tumor types.

The improved therapeutic efficacy observed with DAB-Lf dendriplexes treatment resulted in an extended survival of, respectively, 21 and 16 days for PC-3 and DU145 tumors, compared to untreated tumors (Figures 3(d) and 4(d)).

## Discussion

The possibility of using gene therapy for the treatment of prostate tumors and their metastasis is limited by the inability of the current gene delivery systems to specifically reach their target once intravenously injected, causing secondary effects to healthy tissues. In order to overcome this issue, we hypothesize that a Lf-bearing DAB dendrimer complexed to plasmid DNA encoding TNFα, TRAIL, and IL-12 would enhance the delivery of therapeutic DNA to prostate tumors and increase its therapeutic efficacy *in vitro* as well as *in vivo*.

The conjugation of Lf to DAB dendrimer was achieved by a bioconjugation reaction using GMBS as a cross-linker. GMBS is a heterobifunctional cross-linker with an NHS ester at one end and a maleimide group at the other end. Thus, a two-step synthesis technique was used in order to direct the conjugation reaction towards the selected part of the coupling molecule. The NHS ester was first directed to react with the primary amine group in DAB to form amide linkages, followed by the addition of the targeting ligand. Lf had been modified prior to the reaction with a sulfhydryl group, to react with the maleimide, yielding a thioether bond. The main advantage of this method is that it prevents the polymerization that may occur when using homo-bifunctional cross-linkers. In addition, it offers the highest level of control over the resultant conjugate size by controlling the molar ratio of the reactants at each step (Hermanson, 2013). In contrast to this multi-step method, we previously synthesized DAB-Lf using dimethyl suberimidate as a homo-bifunctional cross-linker (Lim et al., 2015). While this technique presented the advantage of being fast, it yielded compounds whose properties were not closely controlled. Variations may have occurred in the coupling between the reactant compounds, resulting in possible asymmetry in the nanoparticles formed. The stepwise nature of our modified method overcomes this disadvantage.

DAB-Lf was able to condense the negatively charged DNA via electrostatic interactions, although an excess of dendrimer (dendrimer: DNA weight ratios of 5:1 and above) was required to ensure efficient DNA condensation. The DNA condensation observed with DAB-Lf appeared to follow the same pattern as the one described by Kim et al. (2007) and by Aldawsari et al. (2011) when, respectively, using generation 2- and 3-polypropylenimine dendrimers conjugated to positively charged arginine. Both groups demonstrated that the grafting of arginine residues to the dendrimers facilitated the formation of stable dendriplexes due to their strong positive charges at high dendrimer: DNA weight ratios, but reduced the DNA condensing ability of the dendrimer at low dendrimer: DNA ratios, as observed in our current experiment. In addition, DNA condensation was improved compared to the results previously obtained with DAB-Lf synthesized with dimethysuberimidate homo-bifunctional cross-linker: the DNA condensation has increased by at least 10% depending of the dendrimer: DNA weight ratios (Lim et al., 2015).

DAB-Lf dendriplex displayed the required size to access the tumor cells, as the cutoff size for extravasation across tumor vasculature has been found to be 400 nm for most tumors (Yuan et al., 1995). Its size decreased with increasing dendrimer: DNA weight ratios ranging from 0.5:1 until 2:1, and then reached a plateau. This pattern can be explained by the fact that higher ratios of charged dendrimers are more positively charged, therefore more efficient in condensing DNA, which results in the formation of smaller complexes.

Zeta potential experiments demonstrated that DAB-Lf dendriplex was bearing a positive surface charge for most dendrimer: DNA weight ratios, but a negative charge at a dendrimer: DNA weight ratio of 0.5:1, indicating that negatively charged DNA was not condensed yet with the dendrimer at this ratio. This result was consistent with the DNA condensation and the agarose gel electrophoresis results. The positive zeta potential for dendrimer: DNA weight ratio above 0.5:1 is most likely due to the presence of the positively charged amino acids of Lf. It would eventually lead to an increase of the electrostatic interactions of the dendriplexes with negatively charged cellular membranes, resulting in an improved cellular uptake through internalization mechanisms (Mahato et al., 1999). In addition, DAB-Lf dendriplex should avoid nonspecific tissue binding *in vivo*, as its zeta potential is lower than 30 mV (Honary & Zahir, 2013). DAB-Lf therefore has suitable physicochemical properties for being efficient gene delivery systems.

*In vitro*, treatment of the cells with DAB-Lf dendriplex resulted in an enhanced transfection compared with DAB on both the tested cell lines. The transfection results obtained in this study were enhanced compared to those obtained with DAB-DMSI-Lf on other cancer cell lines. DAB-DMSI-Lf: DNA dendriplex resulted in a 1.4-fold higher gene expression compared with unconjugated DAB on A431 and B16F10 cells (Lim et al., 2015), which is less than the 2.1-fold increase in cellular uptake observed in PC-3 cells in the current study. This increased β-gal expression most likely resulted from the enhanced cellular uptake of this dendriplex, as there is a strong correlation between cellular uptake and positive charge density of dendriplexes (Futaki et al., 2001). These data are in line with the flow cytometer results of Wei and colleagues (Wei et al., 2012), who found that the uptake of Lf-bearing liposomes was significantly enhanced by more than double compared with unmodified liposomes in hepatocellular carcinoma. DAB-Lf was also found to increase the uptake of fluorescein-labeled DNA by 2.1-fold compared with unmodified DAB in bEnd.3 brain cells (Somani et al., 2015).

The cellular uptake of fluorescein-labeled DNA complexed to DAB-Lf was partially inhibited by free Lf, phenylarsine oxide, filipin, colchicine, and poly-L-lysine.

Pre-treatment of cells with free Lf led to competition between DAB-Lf dendriplex and free Lf for binding to Lf receptors, suggesting that the internalization of the DNA complexed to DAB-Lf is partly due to Lf receptors- mediated endocytosis. This result is in line with previous publications examining the effect of free Lf on the uptake of modified nanomedicines in various cancer cell lines (Lim et al., 2015).

Both phenylarsine oxide and filipin are pinocytosis inhibitors: phenylarsine oxide has been reported to inhibit clathrin-mediated endocytosis (Visser et al., 2004), while filipin blocks the caveolae-mediated process in clathrin-independent endocytosis (Kim et al., 2007b). Colchicine is known to inhibit macropinocytosis (Liu & Shapiro, 2003), which provides nonspecific endocytosis of macromolecules, whereas cationic poly-L-Lysine acts as an uptake inhibitor for cationic delivery systems.

The cellular uptake of DNA complexed to DAB-Lf was therefore mainly related to clathrin-mediated endocytosis, which is a requisite for Lf receptor-mediated endocytosis, caveolae-mediated endocytosis, but not macropinocytosis. This was in accordance with a previous study by Jiang and colleagues (Jiang et al., 2011), who demonstrated that Lf could be taken up by colorectal cancer cells via clathrin-mediated endocytosis. These results therefore confirm the involvement of Lf receptor-mediated endocytosis and other nonspecific mechanisms in the cellular internalization of DNA complexed to DAB-Lf.

The conjugation of Lf to DAB increased the *in vitro* antiproliferative activity of the dendriplex in the two tested prostate cancer cell lines. The most efficacious treatment observed in this study was DAB-Lf dendriplex expressing TRAIL on DU145 cells. These results correlated with the improved gene expression efficacy following treatment with DAB-Lf dendriplexes. We could not find any studies describing the anti-proliferative effect of Lf-bearing gene-based nanomedicines on prostate cancers to allow a comparison with our results. However, our results were in accordance with previously published viral-based works. Kaliberov and colleagues (2004) have demonstrated that an adenoviral delivery system encoding TRAIL under the control of the vascular endothelial growth factor receptor FLT1 promoter was able to increase DU145 cell death compared with the PC-3 cell line, following a similar trend as the one observed in our study. In our experiments, the IC_50_ resulting from the treatment of the cells by DAB-Lf dendriplex encoding TNFα was higher than those of TRAIL and IL-12 in DU145 cells, despite being the lowest in PC-3 cells. This might be due to the different sensitivity of PC-3 and DU145 prostate cell lines to the Fas ligand, as Fas-sensitive PC-3 cell line was recently shown to be more sensitive to TNFα-mediated apoptosis than Fas-resistant DU145 cell line (Rokhlin et al., 1997). These results, in line with our observations, suggested that TNFα-mediated apoptosis in prostate cancer cells might be determined by factors common in both TNFα and Fas pathways.

*In vivo*, we demonstrated that novel intravenously administered DAB-Lf dendriplex encoding TNFα led to tumor eradication of 70% of PC-3 and 50% of DU145 tumors. Furthermore, treatment also led to tumor eradication of 40% of PC-3 tumors and 20% of DU145 tumors completely disappeared following treatment with DAB-Lf dendriplex encoding TRAIL. To our knowledge, it is the first time that the intravenous administration of Lf-bearing gene-based nanomedicines to mice bearing prostate tumors was able to lead to tumor regression and even complete tumor suppression in some cases. We previously demonstrated that DAB-Tf dendriplexes encoding TNFα, TRAIL, and IL-12 could result in prostate tumor regression (Al Robaian et al., 2014), but their therapeutic efficacy was lower than with DAB-Lf dendriplexes (respectively, 60% and 10% PC-3 tumor suppression following treatment with DAB-Tf dendriplexes encoding TNFα and TRAIL, but 70 and 40% tumor suppression when replacing Tf by Lf). In addition, DAB-Lf dendriplex encoding TNFα had the same therapeutic outcome as DAB-Tf dendriplex on DU145 tumors (50% tumor suppression). DAB-Lf dendriplexes encoding TRAIL and IL-12 also led to 20% DU145 tumor suppression, which was not the case following treatment with DAB-Tf dendriplexes. Other studies have also demonstrated the ability of TNFα, TRAIL, and IL-12-encoding DNA to induce a therapeutic effect on prostate tumors, but using different modalities of treatment: intra-tumoral injection, use of a virus as a delivery system or co-treatment with mifepristone (Gabaglia et al., 2010), radiotherapy (Fujita et al., 2007), oncolytic herpes simplex viruses (Varghese et al., 2006), adenoviral vector-mediated Herpes Simplex Virus/thymidine kinase and ganciclovir (Nasu et al., 2001). These studies mainly showed a slowdown of prostate tumor growth, rather than the tumor regression or suppression observed in some instances in our experiments.

*In vivo* experiments were carried out on mice bearing subcutaneous tumors instead of tumors implanted in the prostate, as the antitumoral activity of the treatment had first to be assessed by monitoring the tumor size by caliper measurement, which requires solid, palpable, and vascularized subcutaneous tumors. As the intravenous administration of targeted dendriplexes on nude athymic male mice bearing subcutaneous tumors resulted in tumor regression, the follow-up experiments will be conducted on mice bearing orthotopically implanted tumors.

In our study, the most potent therapeutic effects were obtained following treatment of PC-3 tumors with DAB-Lf dendriplex encoding TNFα, unlike what was observed in the *in vitro* anti-proliferative assay. This discrepancy could be explained by the fact that TNFα exerts its anti-tumor effects *in vivo* via the death receptor-dependent apoptotic pathway, like TRAIL, but also via its anti-angiogenic effects, that are critical for its anti-cancer activity (Mocellin et al., 2005; Mahalingam et al., 2009). In addition, these tumor-targeted nanomedicines were intravenously injected to the mice, which would allow them to reach metastases and remote tumors, an advantage particularly important in the treatment of prostate tumors.

## Conclusions

We have demonstrated for the first time that a Lf-bearing dendrimer-based gene delivery system complexed to a plasmid DNA encoding TNFα, TRAIL, or IL12 can lead to tumor suppression after intravenous administration.

*In vitro*, the conjugation of Lf to DAB resulted in a significant improvement of the anti-proliferative activity of the dendriplex by up to 5.8-fold in PC-3 cells compared to the unmodified dendriplex.

*In vivo*, the intravenous injection of DAB-Lf dendriplex encoding TNFα led to a tumor suppression for 70% of PC-3 and 50% of DU145 tumors, while treatment with DAB-Lf dendriplex encoding TRAIL led to tumor suppression of 40% of PC-3 tumors and 20% of DU145 tumors. DAB-Lf dendriplex encoding IL12 also resulted in tumor suppression for 20% of both types of prostate tumors, with long term survival of the mice. By contrast, all the tumors treated with the dendrimer, with naked DNA or left untreated were progressive for both tumor types. The animals did not show any signs of toxicity.

Lf-bearing DAB dendriplexes encoding TNFα, TRAIL, and IL-12 therefore hold great potential as a novel approach for the gene therapy of prostate cancer.

## Supplementary Material

IDRD_Duf_s_et_al_Suppemental_Content.docx
