# Supplementary material for: Regression of prostate tumors after intravenous administration of lactoferrin-bearing polypropylenimine dendriplexes encoding TNF-α, TRAIL, and interleukin-12
Source: Drug Deliv. 2018 Mar 1;25(1):679–89. doi: 10.1080/10717544.2018.1440666 (PMC6058574; doi:10.1080/10717544.2018.1440666)
Supplement: IDRD_Duf_s_et_al_Suppemental_Content.docx [file IDRD_A_1440666_SM0316.docx]

Regression of prostate tumors after intravenous administration of lactoferrin-bearing polypropylenimine dendriplexes encoding TNF-α, TRAIL and interleukin-12

(Supplementary material)

**Supplementary methods**

*Conjugation of lactoferrin to DAB*

Generation 3- diaminobutyric polypropylenimine dendrimer (DAB) was conjugated to lactoferrin with GMBS as a cross-linking agent, using a method adapted from Hermanson (Hermanson, 2013) (Supplementary Figure 1). DAB (20 mg) was dissolved in 2 mL of 50 mM sodium phosphate and 0.15 M sodium chloride buffer (pH 7.4). GMBS cross-linker (2 moles excess over DAB, 6.6 mg) reacted with the DAB solution for one hour at 25 ^o^C with continuous stirring. The final compound was filtered by dialysis using benzoylated dialysis tubing with a pore size of 2 000 MWCO for 24 h at 20 ^o^C, using 500 mL of 50 mM sodium phosphate and 0.15 M sodium chloride buffer as the dialysis solution which was changed twice during the dialysis.

The tumor-targeting ligand lactoferrin (Lf) was first modified to hold a sulfhydryl group, to react with the maleimide group of GMBS cross-linker. To do so, 20 mg of Lf was dissolved in 2 mL of 50 mM sodium phosphate and 0.15 M sodium chloride buffer (pH 8) and reacted with 10-fold mole excess of 2-iminothiolane (Traut’s reagent, 2 mg/mL in distilled water, 14.5 mM, 153 µL) for one hour at 20 ^o^C. The modified Lf was isolated using a Vivaspin-4 centrifuge tube with cut-off of 5 000 MWCO for 15 minutes at 6288.75 g and directly conjugated to DAB-maleimide at 25 ^o^C for two hours whilst stirring. The final product was purified using the Vivaspin-4 centrifuge tube in the same conditions as described above, to remove any unreacted dendrimer, followed by a desalting procedure using a dialysis tube with pore size of 3 500 MWCO (SnakeSkin^®^) for 24h at 20 ^o^C, against 1L distilled water, changed once, before being freeze-dried.

The grafting of Lf to DAB was assessed by ^1^H NMR spectroscopy (Jeol Oxford NMR AS 600 spectrometer, Peobody, MA) and MALDI-TOF mass spectroscopy (Axima CFR, Kratos, Shimadzu, Kyoto, Japan).

**
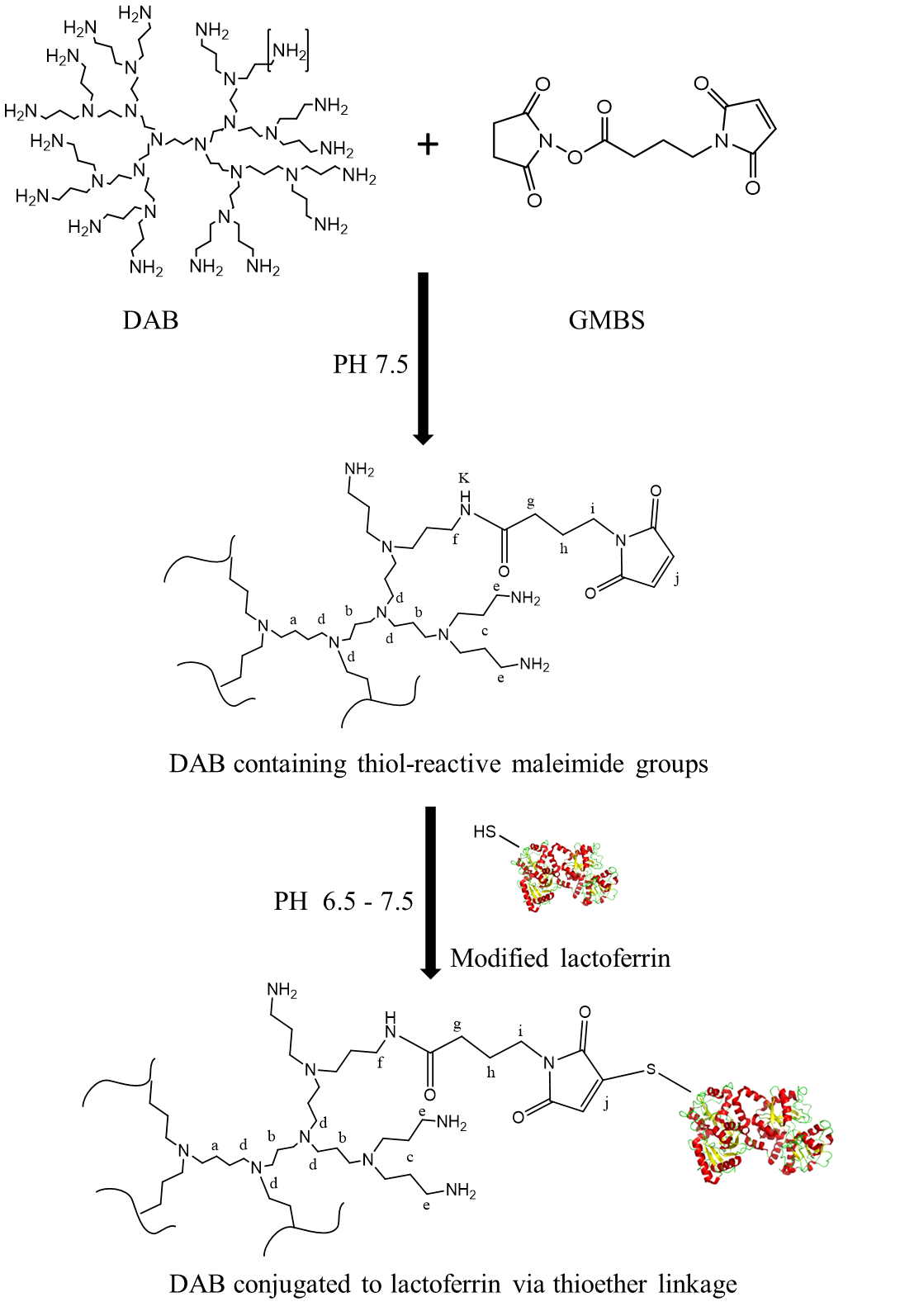
**

**Supplementary Figure 1.** Conjugation of lactoferrin to the dendrimer DAB via thioether linkage

*Characterization of DAB-Lf-DNA dendriplex formation*

The ability of DAB-Lf dendrimer to successfully complex plasmid DNA was assessed by PicoGreen^®^ assay, following the protocol from the supplier. PicoGreen^®^ reagent was diluted 200-fold in Tris-EDTA (TE) buffer (10 mM Tris, 1mM EDTA, pH 7.5) before the experiment. One mL of DAB-Lf: DNA dendriplex at various dendrimer: DNA weight ratios (20:1, 10:1, 5:1, 2:1, 1:1, 0.5:1 and 0:1) was added to one mL of the diluted PicoGreen^®^ reagent. The DNA concentration in the cuvette (10 µg/mL) remained constant throughout the whole experiment. The fluorescence intensity of PicoGreen^®^ in the presence of the formed dendriplexes was measured at various times using a Varian Cary Eclipse Fluorescence spectrophotometer (Palo Alto, CA) (λ_exc_: 480 nm, λ_em_: 520 nm). Results were presented as the percentage of DNA condensation.

The ability of DAB-Lf conjugate to complex with DNA was also assessed by agarose gel retardation assay. DAB-Lf dendriplexes were prepared at various dendrimer: DNA weight ratios from 20:1 to 0.5:1, with a constant DNA concentration of 20 µg/mL. After mixing with the loading buffer, the samples (15 µL) were loaded on a 1X Tris-Borate-EDTA (TBE) (89 mM Tris base, 89 mM boric acid, 2 mM Na_2_-EDTA, pH 8.3) buffered 0.8% (w/v) agarose gel containing ethidium bromide (0.4 µg/mL), with 1x TBE as a running buffer. The DNA size marker was HyperLadder I. The gel was run at 50 V for 1h and then photographed under UV light.

Nanoparticles of DAB-Lf complexed with DNA were also visualized by atomic force microscopy (AFM) to assess their size and shape. DAB-Lf (250 µg) was complexed with 50 µg plasmid DNA encoding β-galactosidase at a dendrimer: DNA weight ratio of 5:1, using glucose 5% solution as a solvent. Following 100-fold dilution with distilled water, 5 µL of the diluted complex was placed on a mica surface and left to dry at 25 ^o^C. The scanning mode used was PeakForce Tapping^®^ at room temperature, using a ScanAsyst-Air^®^ probe with nominal tip radius of 2 nm and nominal spring constant of k = 0.4 N/m (Bruker, Billerica, MA). Data were collected by a Dimension Icon^®^ AFM (Bruker).

*Dendriplex size and zeta potential measurement*

The size and zeta potential of the DAB-Lf dendriplexes were measured for the different dendrimer: DNA weight ratios (20:1, 10:1, 5:1, 2:1, 1:1, 0.5:1, 0:1) by photon correlation spectroscopy and laser Doppler electrophoresis using a Malvern Zetasizer Nano-ZS (Malvern Instruments, Malvern, UK).

**Supplementary results**

***Synthesis and characterization of lactoferrin-bearing DAB***

*Conjugation of lactoferrin to DAB*

^1^H-NMR of DAB-cross-linker (Supplementary Figure 2) confirmed the conjugation between DAB and GMBS as follows: ^1^H-NMR (D_2_O): δ DAB (N-CH_2_-**CH_2_-CH_2_**-CH_2_-N) = 1.48 (a); δ DAB (N-CH_2_-**CH_2_**-CH_2_-N) = 1.64 (b); δ DAB (N-CH_2_-**CH_2_**-CH_2_-NH_2_) = 1.79 (c); δ DAB (N-CH_2_-CH_2_-**CH_2_**-NH) = 2.48 (f); δ DAB (N-**CH_2_**-CH_2_-**CH_2_**-N) = 2.55 (d); δ DAB (N-CH_2_-CH_2_-**CH_2_**-NH_2_) = 2.91 (e). The peaks corresponding to the cross-linker protons in the same spectrum were as follows: protons for carbon (h) at 2.21 ppm; for carbon (g) at 2.98 ppm; for carbon (i) at 3.15 ppm; maleimide group protons (j) at 6.23 ppm; amide proton for N (k) at 8.05 ppm.

The synthesis of DAB-Lf was confirmed by ^1^H-NMR (Supplementary Figure 2) and MALDI-TOF mass spectroscopy (Supplementary Figure 3). ^1^H-NMR showed the peaks corresponding to DAB-cross-linker in the region from 1.5 to 3.5 ppm, as well as peaks corresponding to Lf moieties at 0.8 ppm (l), 3.5 ppm (m) and 8.5 (n). MALDI-TOF spectrum also showed the average molecular weight of DAB-Lf at a peak of 84 537.5 m/z compared with Lf alone at a peak of 82 751.31 m/z.


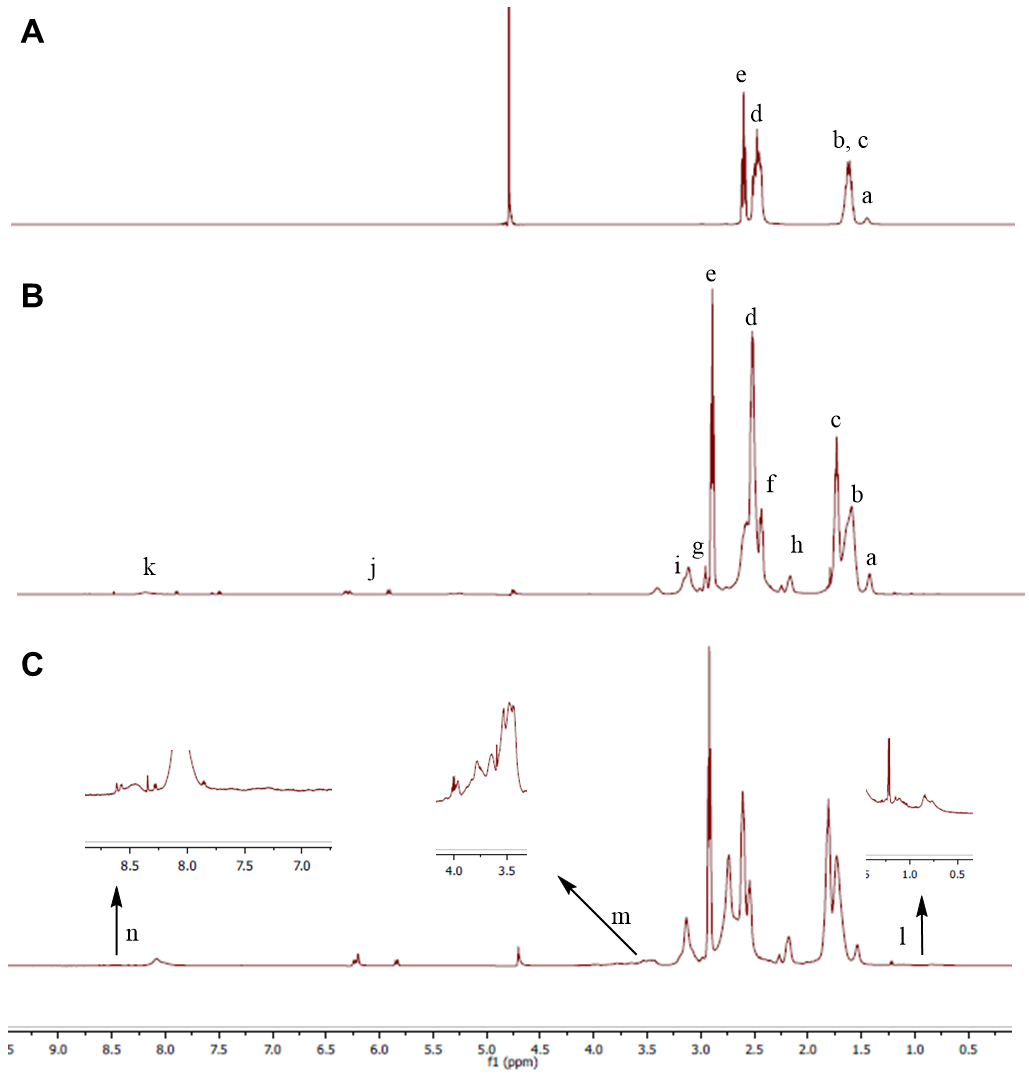


**Supplementary Figure 2.** ^1^H NMR spectra of DAB (A), DAB-crosslinker (B) and DAB-Lf (C)


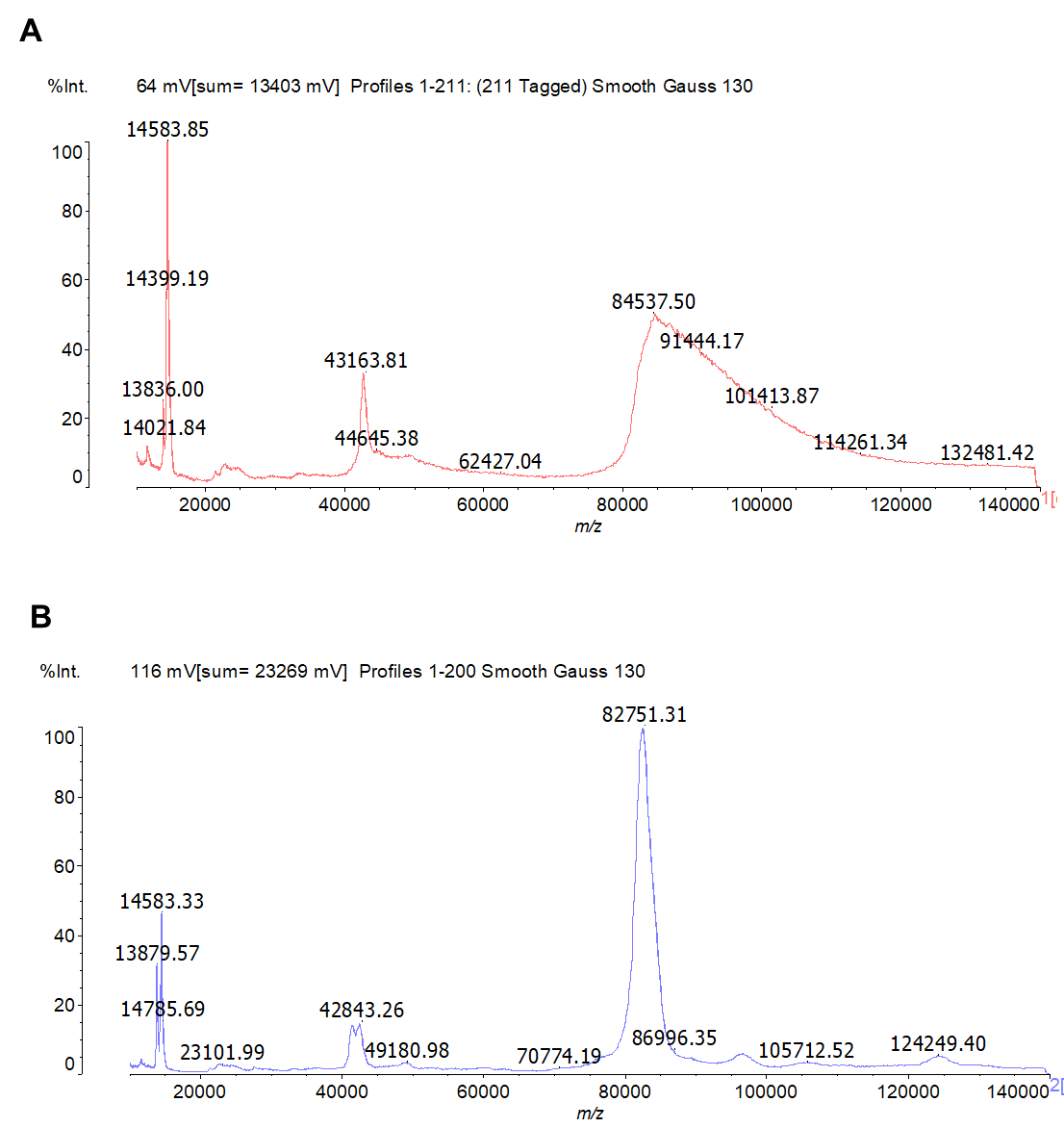


**Supplementary Figure 3.** MALDI-TOF spectra of DAB-Lf (A) and Lf only (B)

*Characterization of DAB-Lf-DNA dendriplex formation*

DAB-Lf was able to condense more than 70% of the DNA at dendrimer: DNA weight ratios of 5:1 and higher (Supplementary Figure 4A). DNA condensation occurred instantly and was found to be stable for at least 24 h. It increased with increasing weight ratios and reached a maximum of 80% at a dendrimer: DNA weight ratio of 20:1. The DNA condensation observed for dendrimer: DNA weight ratios of 2:1 or higher was much higher than that observed for the unmodified dendrimer, which was previously shown to be 60% at its best and decreasing with time (Aldawsari *et al*., 2011).

A gel retardation assay confirmed the complete and partial DNA condensation by DAB-Lf dendrimer (Supplementary Figure 4B). At dendrimer: DNA weight ratios of 20:1, 10:1 and 5:1, DNA was fully condensed by DAB-Lf, thus preventing ethidium bromide to intercalate with DNA. No free DNA was therefore visible at this ratio. In contrast, DNA was partially condensed by DAB-Lf dendrimer for the dendrimer: DNA ratios lower than 5:1. Ethidium bromide could therefore intercalate with DNA and a band corresponding to free DNA was visible.

The formation of spherical nanoparticles of DAB-Lf complexed to DNA was also demonstrated by atomic force microscopy (Supplementary Figure 4C).

Lactoferrin-bearing DAB dendriplex displayed an average size less than 200 nm at all dendrimer: DNA weight ratios used (Supplementary Figure 4D). Its size decreased with increasing dendrimer: DNA weight ratios ranging from 0.5:1 until 2:1, and then reached a plateau. DAB-Lf dendriplex was found to be the largest at a dendrimer: DNA weight ratio of 0.5:1, with an average size of 200.7 ± 6.8 nm, while it was the smallest at a dendrimer: DNA weight ratio of 20:1, with an average size of 64.3 ± 0.9 nm. These sizes were in accordance with those determined by AFM.

Zeta potential experiments demonstrated that the DAB-Lf dendriplex was bearing an almost steady positive surface charge comprised between 17 and 20 mV for most ratios, but a negative charge of -15 ± 2 mV at a dendrimer: DNA weight ratio of 0.5:1 (Supplementary Figure 4E).


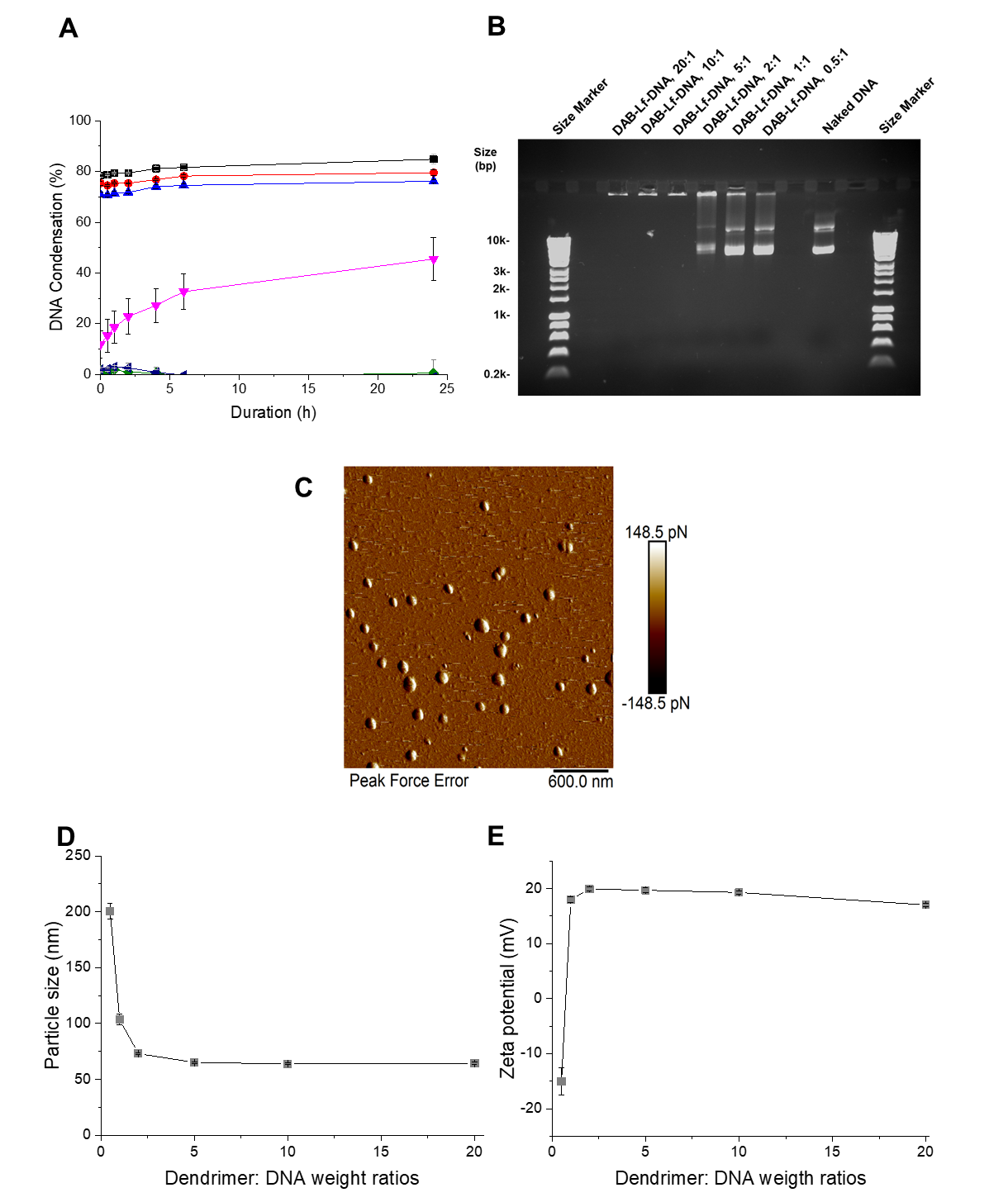


**Supplementary Figure 4.** Characterization of DAB-Lf dendriplex:

A) DNA condensation of DAB-Lf dendriplex using PicoGreen® reagent at various durations and dendrimer: DNA weight ratios: 20:1 (■, black), 10:1 (●, red), 5:1 (▲, blue), 2:1 (▼, pink), 1:1 (♦, green) and 0.5:1 (◄, violet). Results are expressed as mean ± SEM (n= 4).

B) Gel retardation assay of DAB-Lf dendriplex at various dendrimer: DNA weight ratios (20:1, 10:1, 5:1, 2:1, 1:1, 0.5:1).

C) Atomic force microscopy height image of DAB-Lf dendriplex (dendrimer: DNA weight ratio 5:1) (Bar: 600 nm)

D) Size of DAB-Lf dendriplex at various dendrimer: DNA weight ratios (20:1, 10:1, 5:1, 2:1, 1:1, 0.5:1). Results are expressed as mean ± SEM (n= 4).

E) Zeta potential of DAB-Lf dendriplex at various dendrimer: DNA weight ratios (20:1, 10:1, 5:1, 2:1, 1:1, 0.5:1). Results are expressed as mean ± SEM (n= 4).


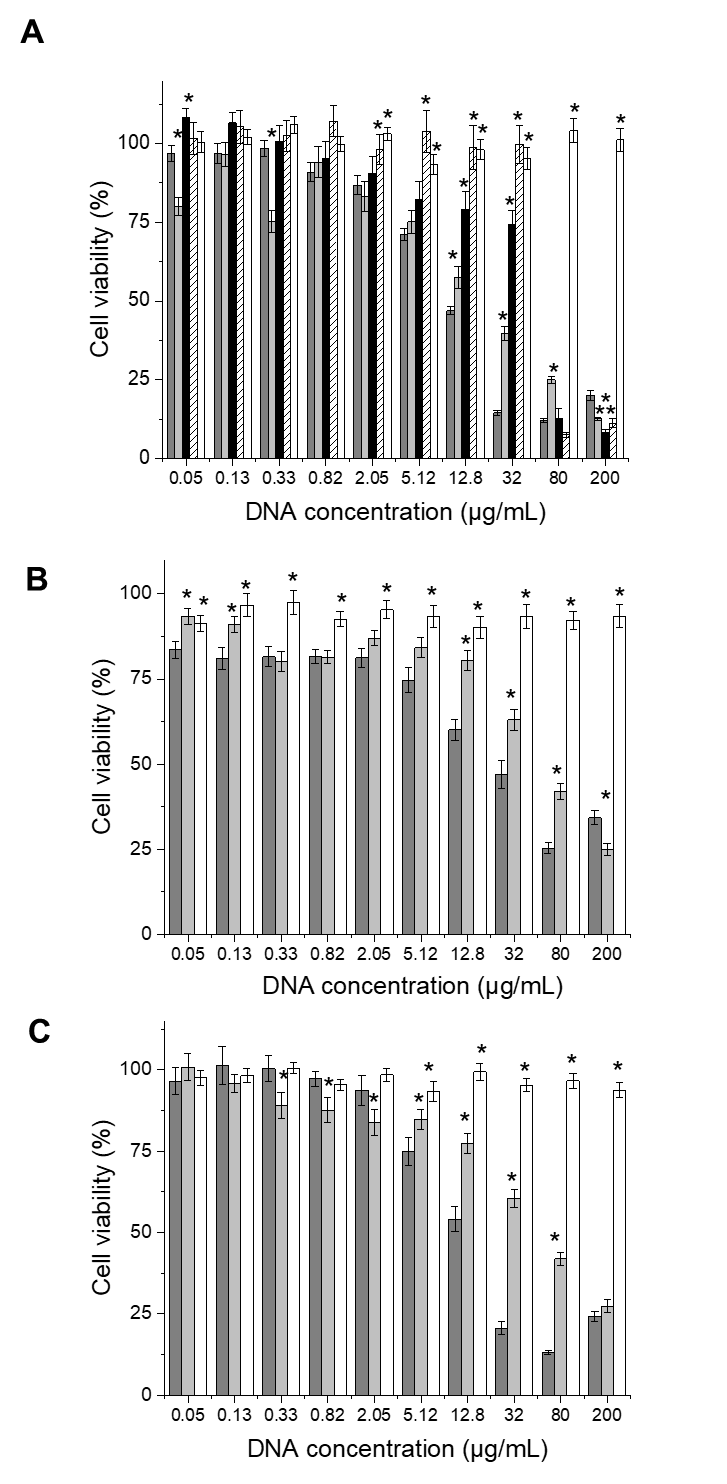


**Supplementary Figure 5.** Anti-proliferative efficacy of DAB-Lf dendriplexes (dark grey) encoding TNFα (A), TRAIL (B) and IL-12 (C), in PC-3 prostate cancer (controls: DAB dendriplexes (light grey), DAB-Lf only (black), DAB only (white with stripes), DNA only (white) (n=15) (*: P <0.05 compared with DAB-Lf-DNA).


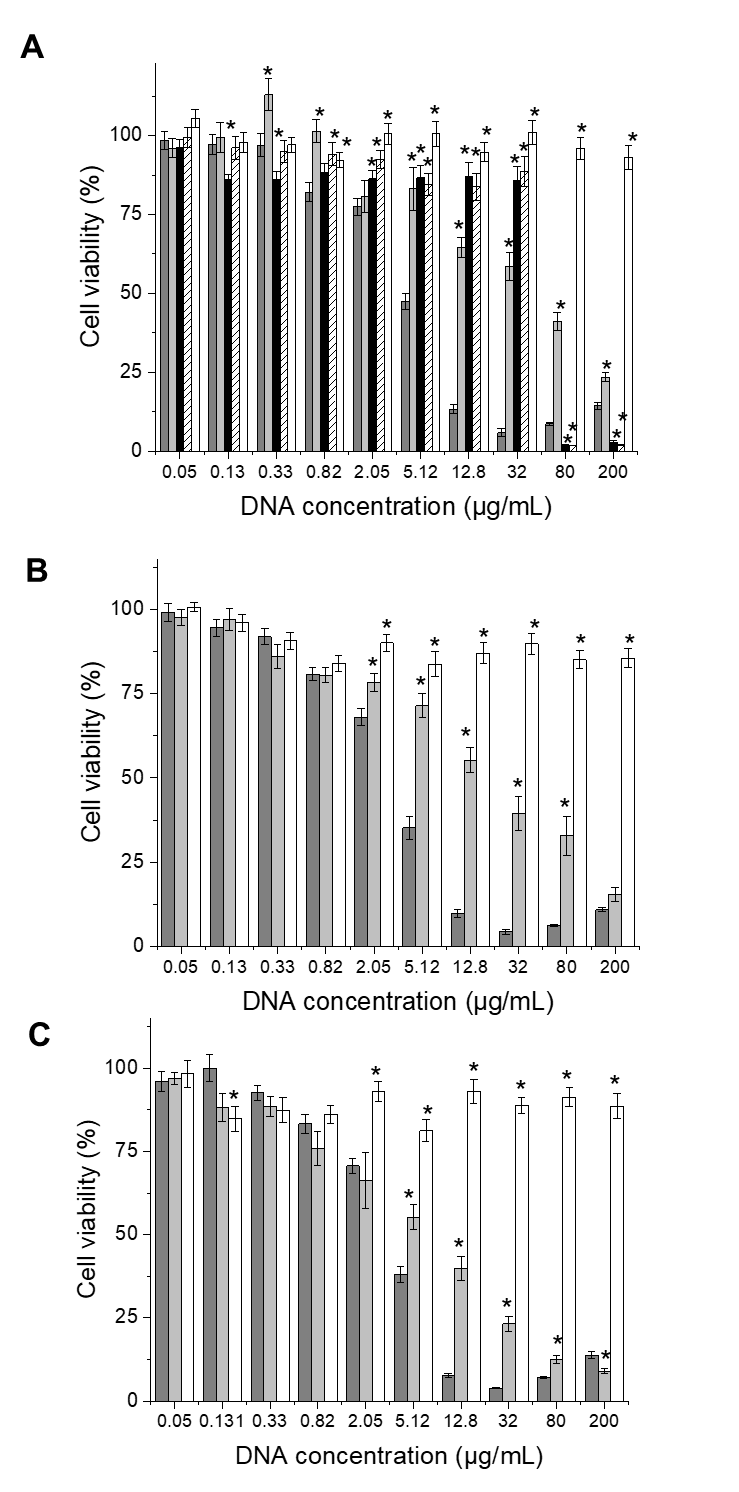


**Supplementary Figure 6.** Anti-proliferative efficacy of DAB-Lf dendriplexes (dark grey) encoding TNFα (A), TRAIL (B) and IL-12 (C), in DU145 prostate cancer (controls: DAB dendriplexes (light grey), DAB-Lf only (black), DAB only (white with stripes), DNA only (white) (n=15) (*: P <0.05 compared with DAB-Lf-DNA).
